# Supplementary figures and images for: Small subpopulations of β-cells do not drive islet oscillatory [Ca2+] dynamics via gap junction communication
Source: PLoS Comput Biol. 2021 May 3;17(5):e1008948. doi: 10.1371/journal.pcbi.1008948 (PMC8118513; doi:10.1371/journal.pcbi.1008948)

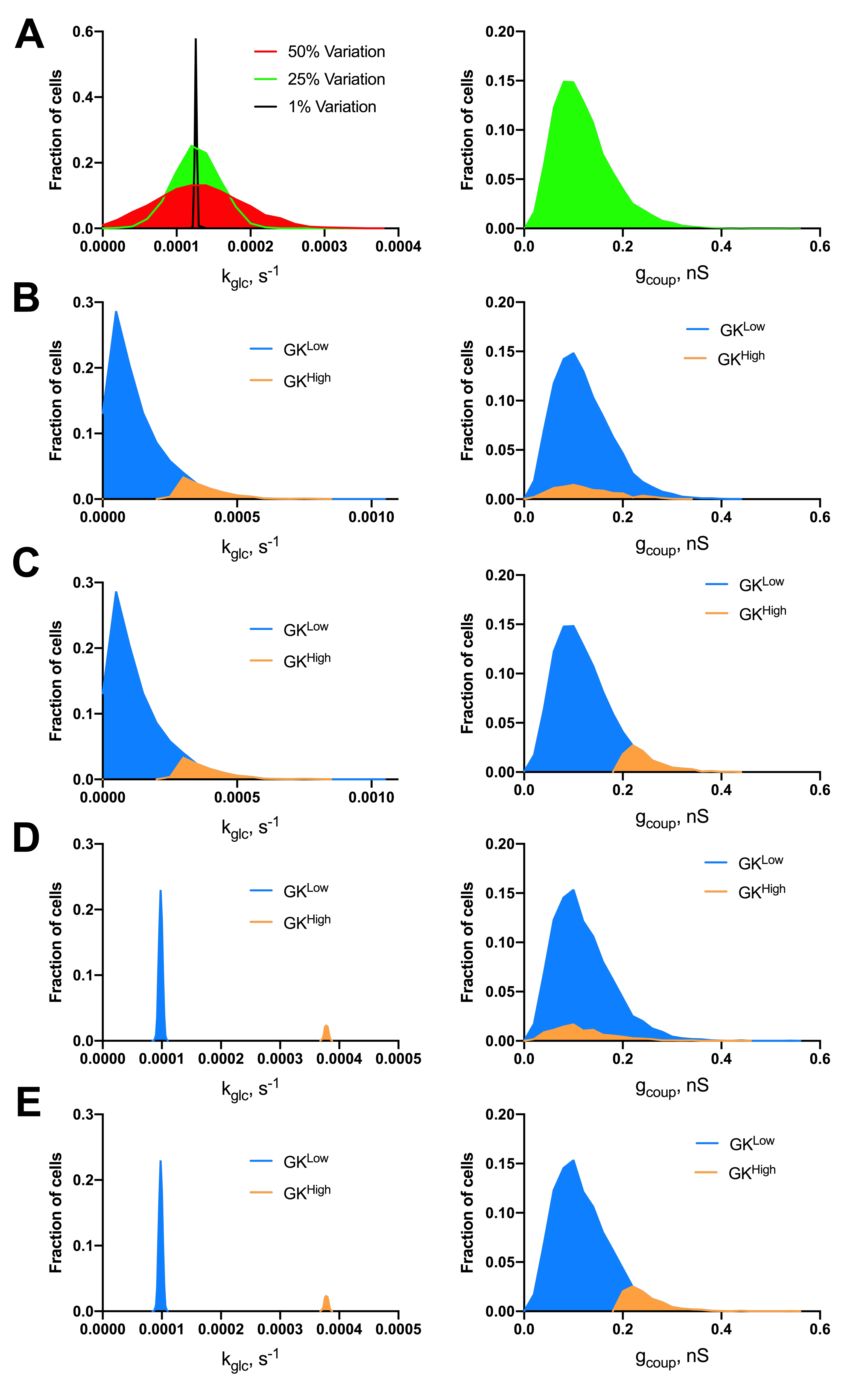

Supplement: S1 Fig — A). All unimodal normal distributions’ histograms. Left: Average frequency of cells at varying GK rate (kglc) for simulations that have different standard deviations in GK activity from Fig 1. Right: Corresponding histogram of average frequency of cells at varying coupling conductance (gCoup). B). As in A but for simulations with a skewed normal distribution of GK activity from Fig 2A–2E. C). As in A but for simulations with a skewed normal distribution of GK activity and correlated GK and gCoup activity from Fig 3A–3C. D). As in A but for simulations with a bimodal distribution of GK activity from Fig 2I–2K. E). As in A for simulations with bimodal distribution of GK activity and correlated GK and gCoup from Fig 3D–3F. Data representative of 5 simulations with differing random number seeds. (TIF) [file pcbi.1008948.s001.tif]

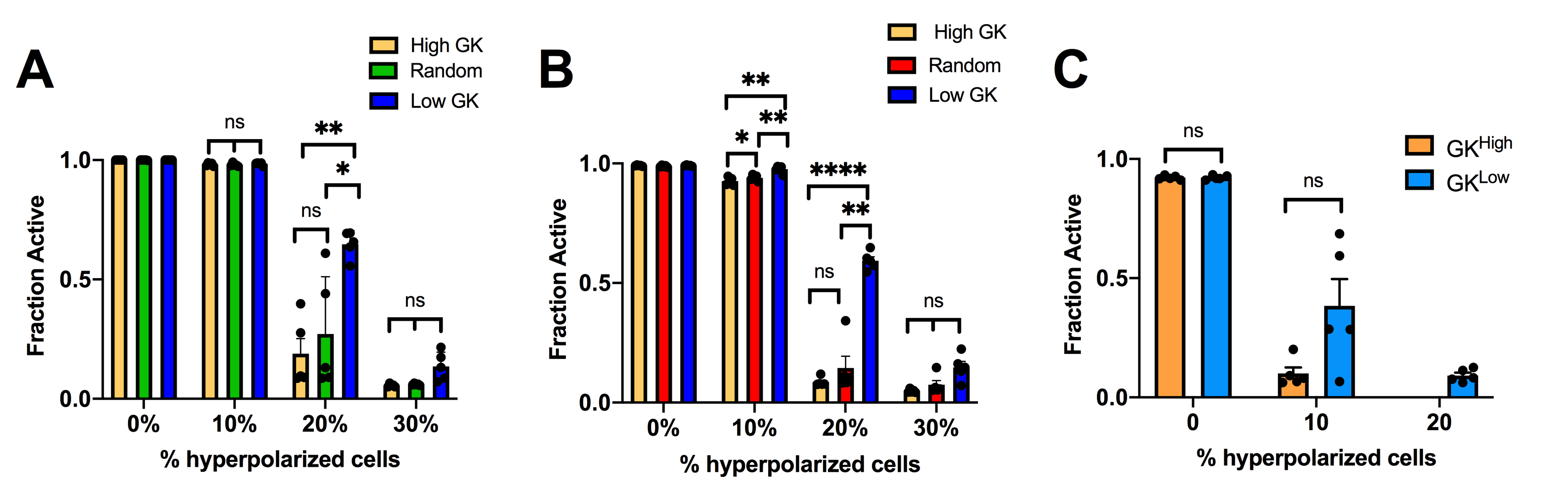

Supplement: S2 Fig — A). Fraction of cells showing elevated [Ca2+] activity (active cells) vs. the percentage of cells hyperpolarized in islet from simulations with a unimodal normal distribution with 25% variation in GK activity (kglc). Simulations run in the presence of stochastic noise (see methods). B). As in A but for simulations with 50% variation in GK activity. C). As in A but for simulations with unimodal skewed distribution. Error bars are mean ± s.e.m. Repeated measures one-way ANOVA with Tukey post-hoc analysis was performed for A and B. Student’s paired t-test was performed to test for significance in C. Significance values: ns indicates not significant (p>.05), * indicates significant difference (p < .05), ** indicates significant difference (p < .01), *** indicates significant difference (p < .001), **** indicates significant difference. Data representative of 5 simulations with differing random number seeds. (TIF) [file pcbi.1008948.s002.tif]

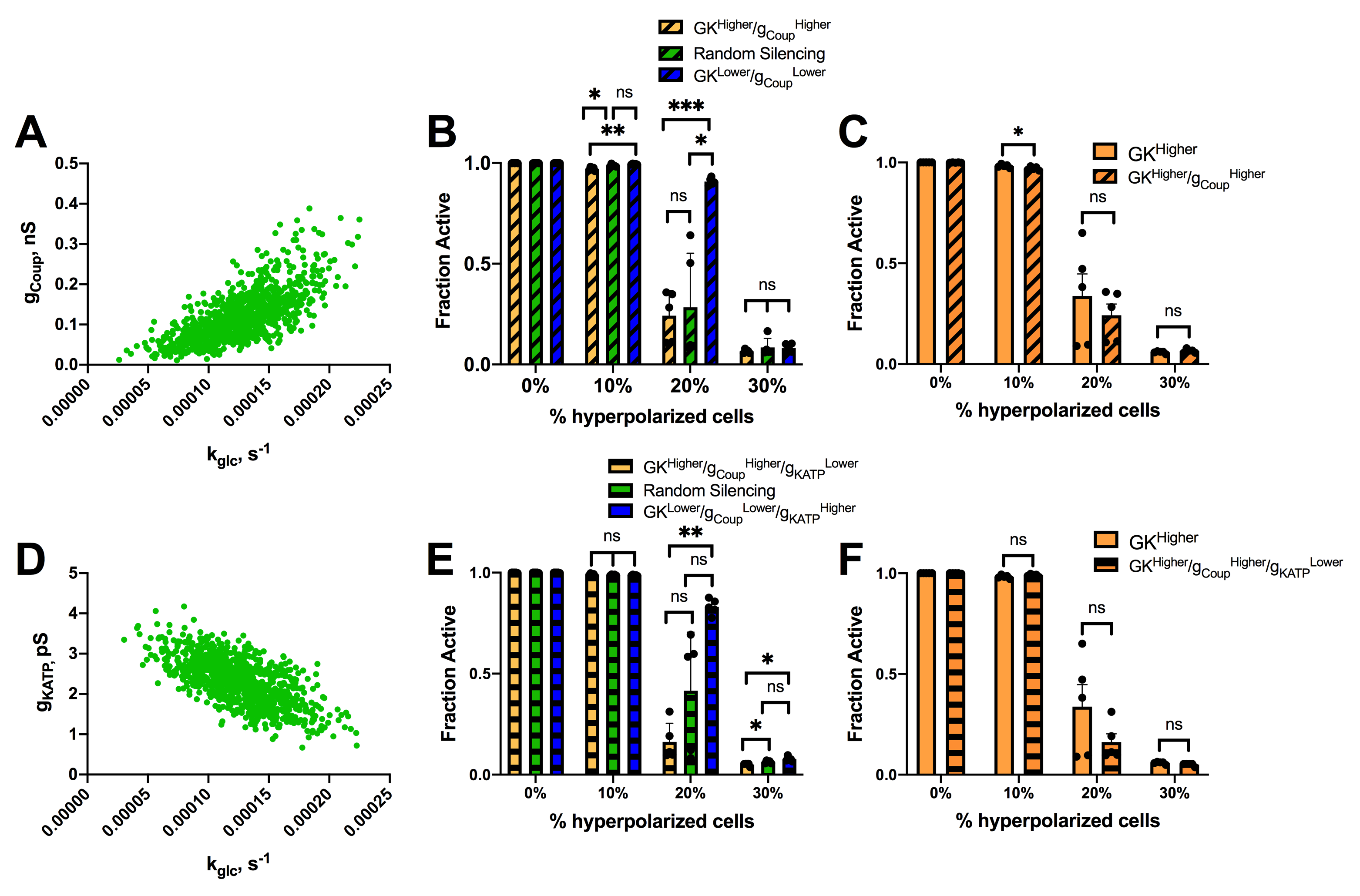

Supplement: S3 Fig — A). Scatterplot of gCoup vs. kglc for each cell from a representative simulation where gCoup is correlated with kglc for simulation where GK activity is a modeled as a unimodal normal distribution. B). Fraction of cells showing elevated [Ca2+] activity (active cells) vs. the percentage of cells hyperpolarized in islet from simulations with a unimodal normal distribution in kglc with correlated gCoup and kglc as in A. Hyperpolarized cells are chosen based on their GK rate which is correlated to gCoup. C). As in B. but comparing hyperpolarization in high GK cells in the presence (B) and absence (Fig 1C) of correlations in gCoup. D). as in A but from a simulation where gCoup and kglc and gKATP (KATP channel conductance) are correlated. E). As in B. but for simulations where gCoup and kglc and gKATP are correlated. F). As in C. but comparing high GK cells hyperpolarization from Fig 1C to high GK hyperpolarization from simulations where gCoup and kglc and gKATP are correlated (E). Error bars are mean ± s.e.m. Repeated measures one-way ANOVA with Tukey post-hoc analysis was performed for simulations in B and C (if there were any missing values a mixed effects model was used) and a Student’s t-test was performed for C and F (Welches t-test for unequal variances was used when variances were determined to be statistically different using an F-test) to test for significance. Significance values: ns indicates not significant (p>.05), * indicates significant difference (p < .05), ** indicates significant difference (p < .01), *** indicates significant difference (p < .001), **** indicates significant difference (p < .0001). Data representative of 5 simulations with differing random number seeds. (TIF) [file pcbi.1008948.s003.tif]

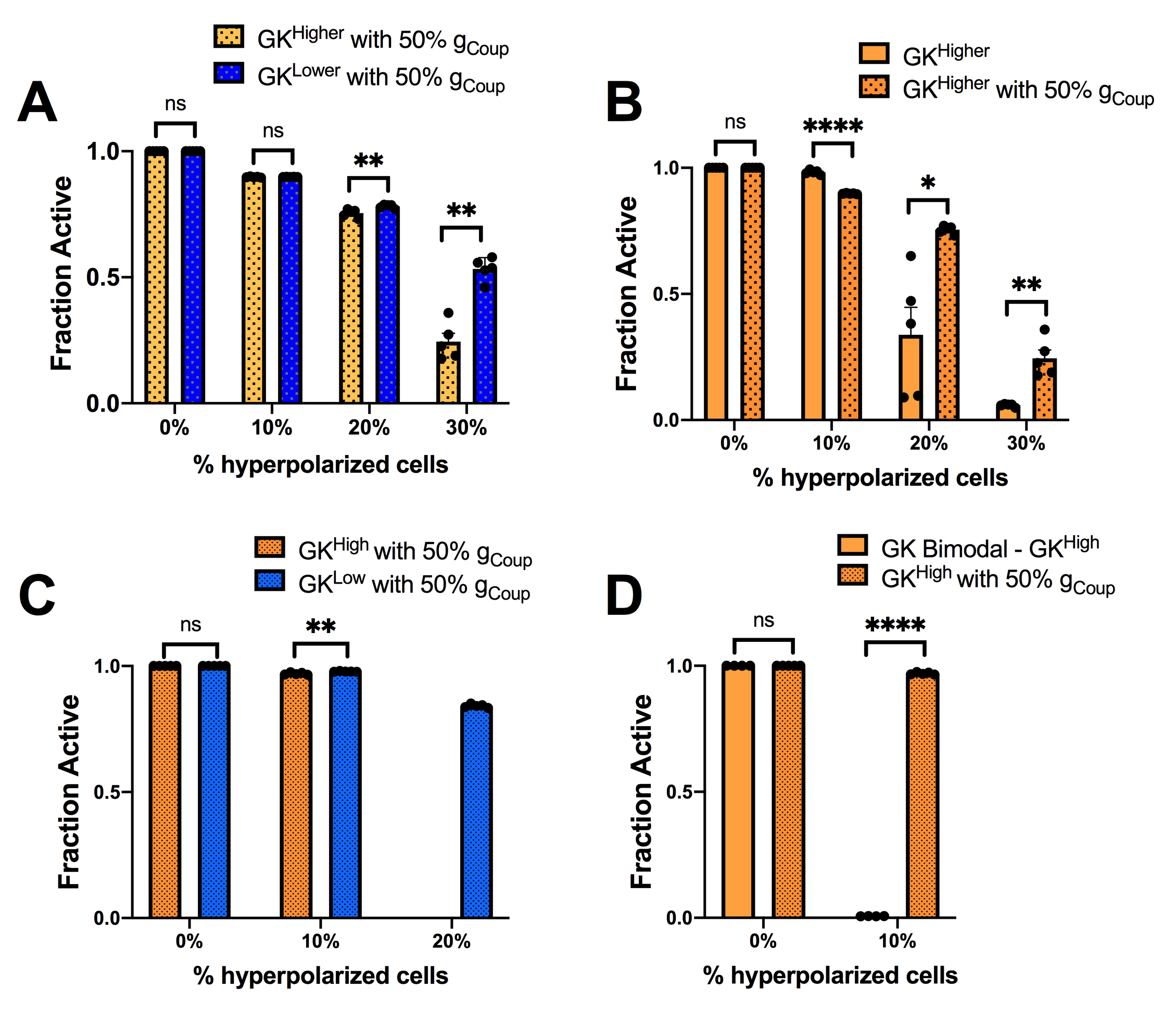

Supplement: S4 Fig — A). Fraction of cells showing elevated [Ca2+] activity (active cells) vs. the percentage of cells hyperpolarized in islet from simulations with a unimodal normal distribution as in Fig 1C but with 50% reduction in average coupling conductance (60pS) for all cells. Hyperpolarized cells are chosen based on their GK rate. B). As in A. but comparing hyperpolarization in high GK cells in simulations with full coupling (120pS–Fig 1C) and reduced coupling (60pS) from A. C). as in A but for bimodal simulations with reduced coupling (60pS). D). As in B but comparing bimodal distributions in GK with full coupling (120pS) from Fig 2J to bimodal simulations with reduced coupling (60pS) from C. Error bars are mean ± s.e.m. Student’s paired t-test was performed to test for significance for all simulations. Significance values: ns indicates not significant (p>.05), * indicates significant difference (p < .05), ** indicates significant difference (p < .01), *** indicates significant difference (p < .001), **** indicates significant difference. Data representative of 4–5 simulations with differing random number seeds. (TIF) [file pcbi.1008948.s004.tif]

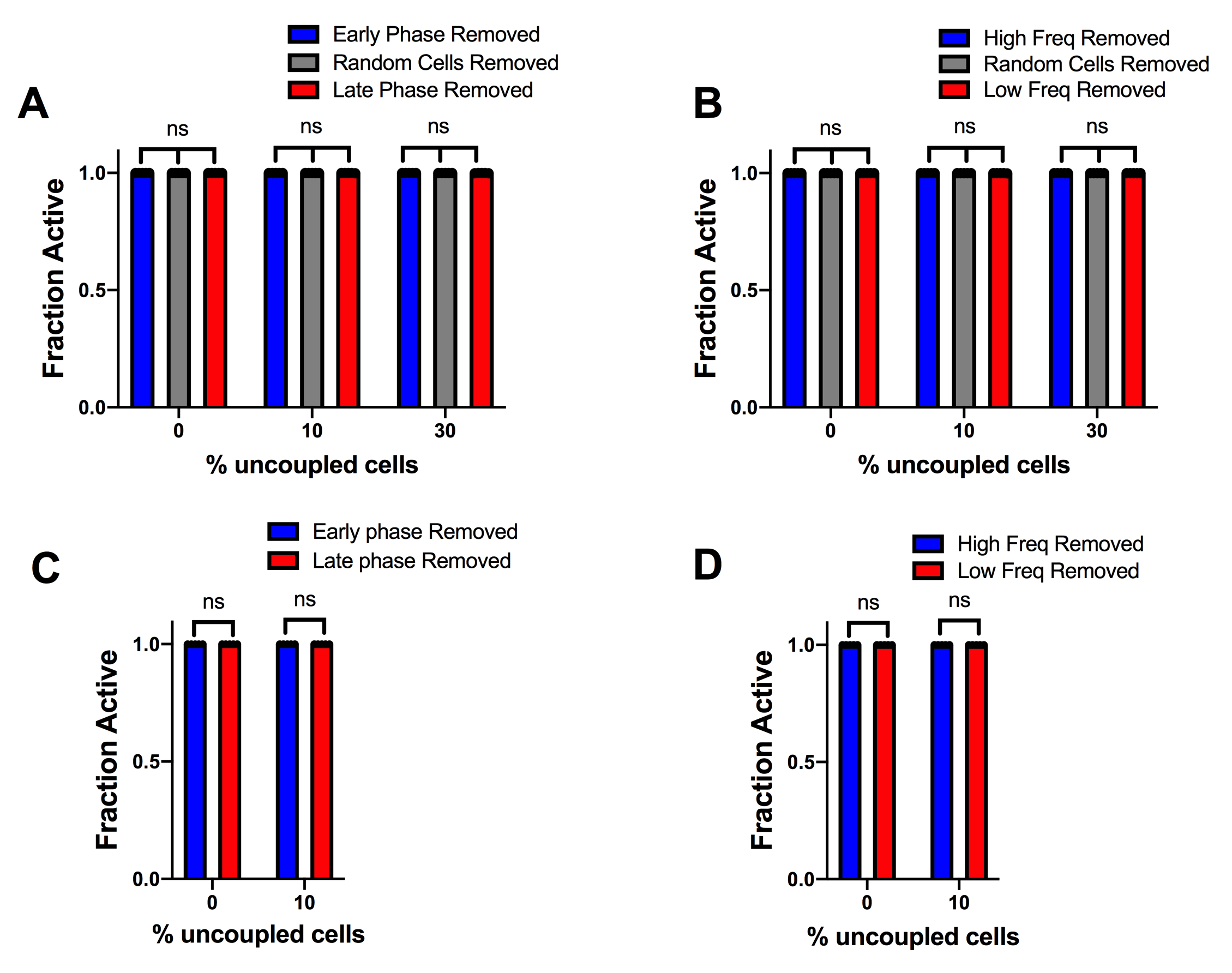

Supplement: S5 Fig — A). Fraction of cells showing elevated [Ca2+] activity (active cells) in simulated islets vs. the percentage of cells uncoupled in islet from simulations in Fig 4. B). As in A but for simulations in Fig 5. C). As in A but for simulations in Fig 6. D). As in A but for simulations in S8 Fig. Error bars are mean ± s.e.m. Repeated measures one-way ANOVA was performed for simulations in A and B and a Student’s paired t-test was performed for C and D to test for significance. Significance values: ns indicates not significant (p>.05), * indicates significant difference (p < .05), ** indicates significant difference (p < .01), *** indicates significant difference (p < .001), **** indicates significant difference. Data representative of 5 simulations with differing random number seeds. (TIF) [file pcbi.1008948.s005.tif]

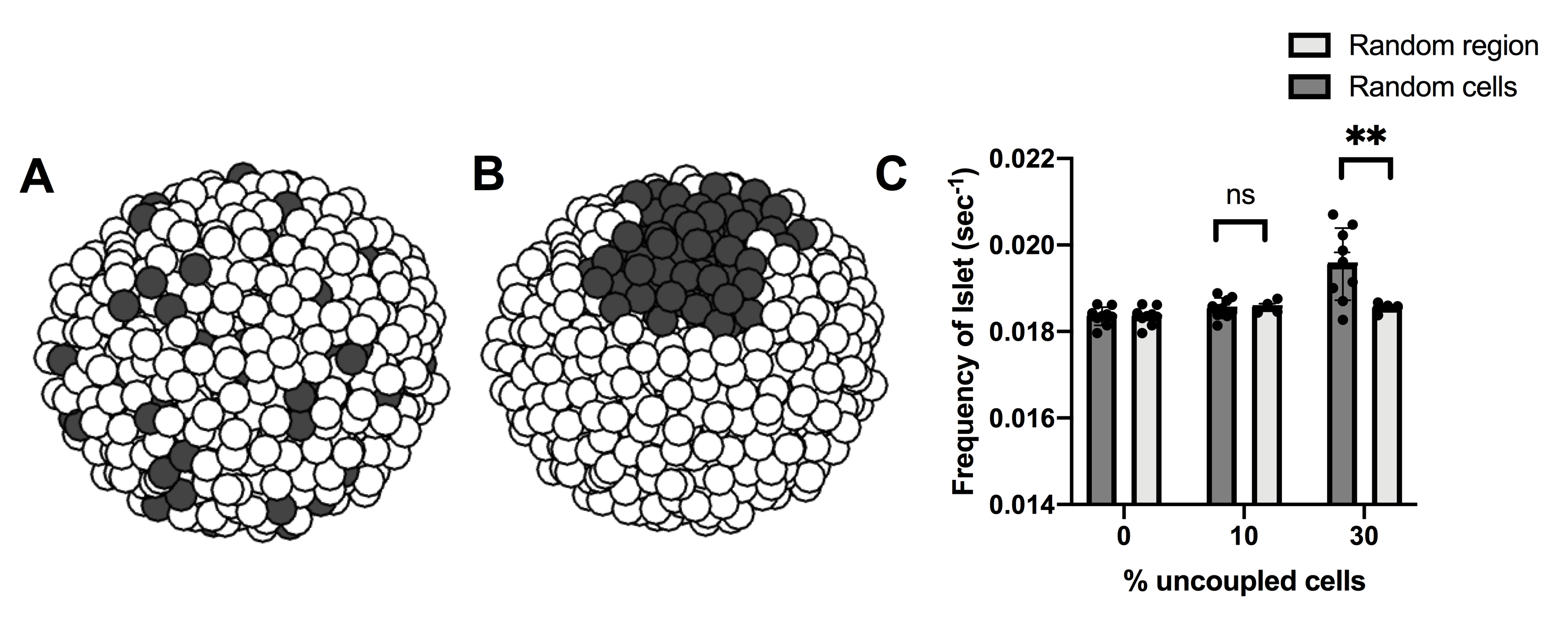

Supplement: S6 Fig — A). Schematic showing which cells are chosen to be removed when a random selection of cells is chosen across the islet. B). Schematic showing which cells are chosen to be removed when a random region of cells is chosen. C). The frequency of the islet after removal of 0%, 10%, or 30% of randomly chosen cells or from a random region. Error bars are mean ± s.e.m. Student’s t-test was performed for 10% and a Welch’s t-test for unequal variances was used to test for significance at 30% of cells removed. Significance values: ns indicates not significant (p>.05), * indicates significant difference (p < .05), ** indicates significant difference (p < .01), *** indicates significant difference (p < .001), **** indicates significant difference. Data representative of 4–9 simulations with differing random number seeds. (TIF) [file pcbi.1008948.s006.tif]

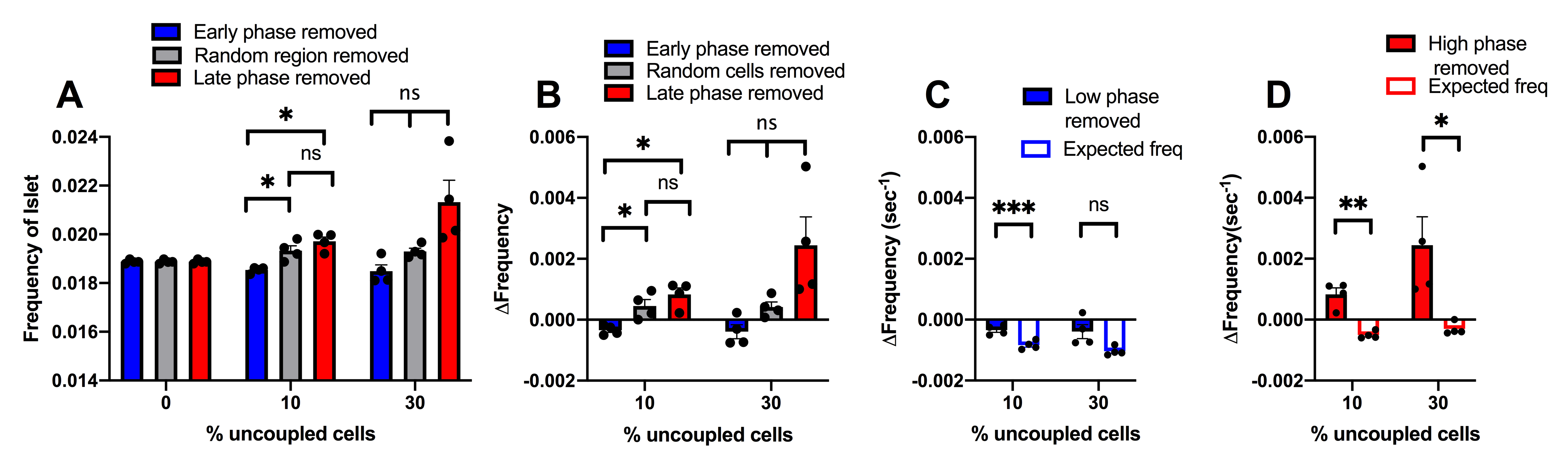

Supplement: S7 Fig — A). Average frequency of islet when indicated populations of cells are removed from the simulated islet with 50% reduction in coupling conductance (60pS). B). Change in frequency of islet with indicated populations removed with respect to control islet with all cells present. C). Change in frequency when early phase cells are removed compared to average oscillation frequency of remaining cells that indicates the expected oscillation frequency. D). Same as C. but for simulations where late phase cells are removed. Error bars are mean ± s.e.m. Repeated measures one-way ANOVA with Tukey post-hoc analysis was performed for simulations in A-B and a Student’s paired t-test was performed for C and D to test for significance. Significance values: ns indicates not significant (p>.05), * indicates significant difference (p < .05), ** indicates significant difference (p < .01), *** indicates significant difference (p < .001), **** indicates significant difference (p < .0001). Data representative of 4 simulations with differing random number seeds. (TIF) [file pcbi.1008948.s007.tif]

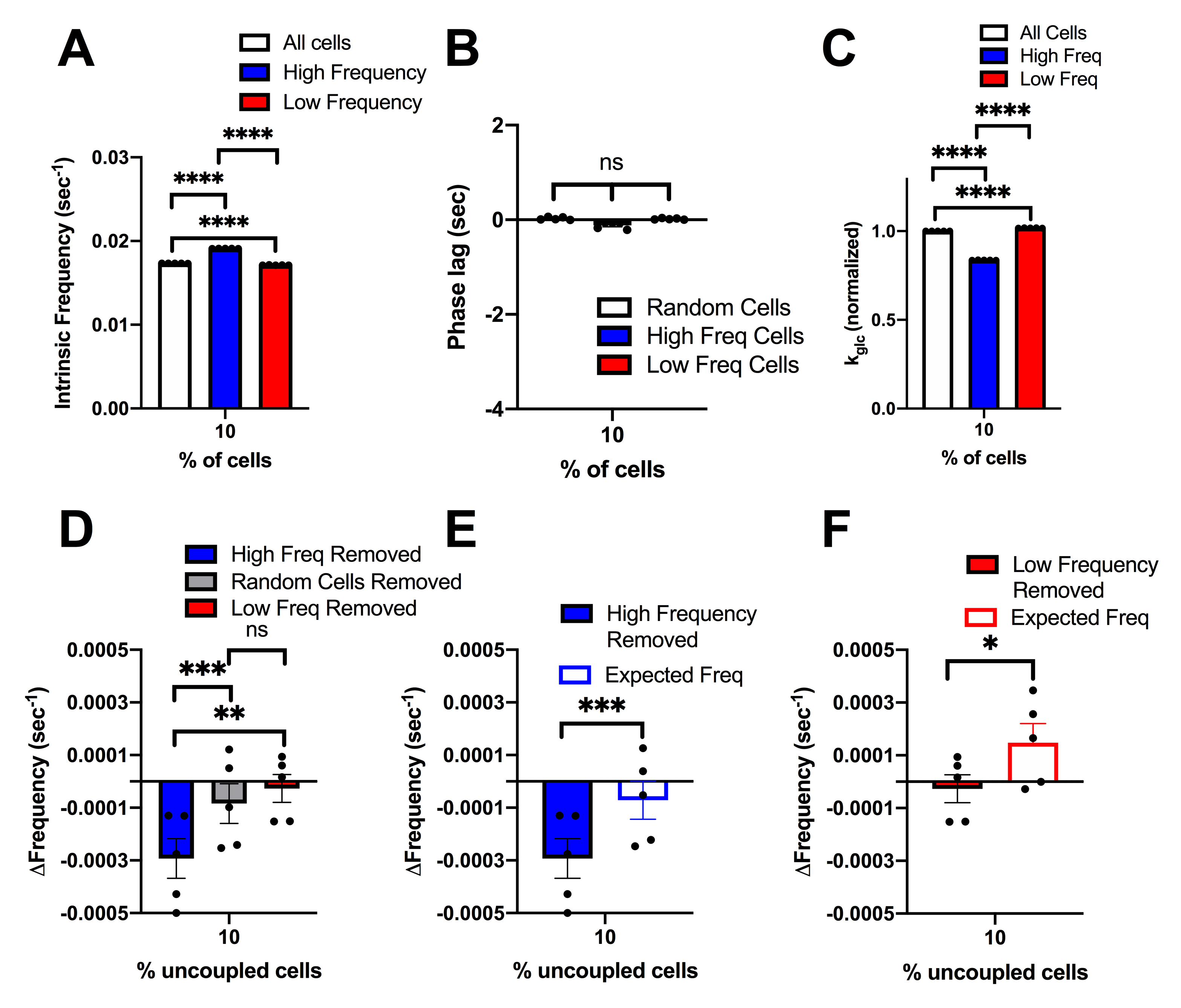

Supplement: S8 Fig — A). Average intrinsic oscillation frequencies of all cells, top 1% or 10% of high frequency cells, or low frequency cells when re-simulated in the absence of gap junction coupling from bimodal model of phase. B). Phase lag from islet average of top 1% or 10% of high frequency, low frequency cells, or random cells. C). Average kglc from all cells, high frequency cells, or low frequency cells across simulated islet. D). Change in frequency of islet with indicated populations removed with respect to control islet with all cells present. E). Change in frequency when high frequency cells are removed compared to average oscillation frequency of remaining cells that indicates the expected oscillation frequency. F). Same as E. but for simulations where low frequency cells are removed. Error bars are mean ± s.e.m. Repeated measures one-way ANOVA with Tukey post-hoc analysis was performed for simulations in A-D and a Student’s paired t-test was performed for E and F to test for significance. Significance values: ns indicates not significant (p>.05), * indicates significant difference (p < .05), ** indicates significant difference (p < .01), *** indicates significant difference (p < .001), **** indicates significant difference (p < .0001). Data representative of 5 simulations with differing random number seeds. (TIF) [file pcbi.1008948.s008.tif]

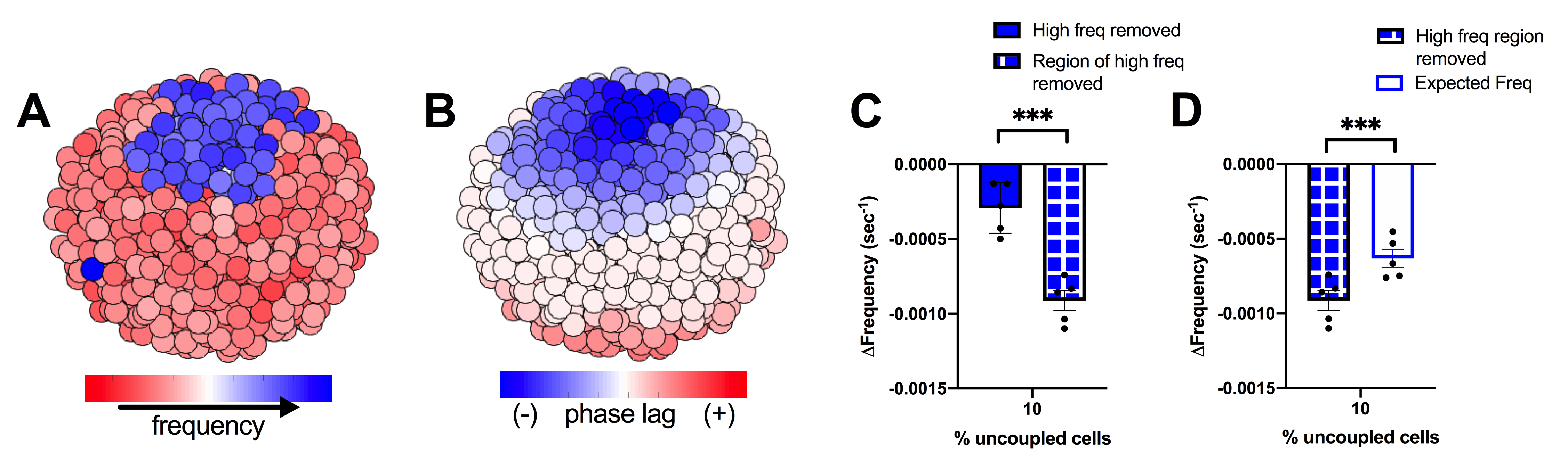

Supplement: S9 Fig — A). Schematic of frequency across simulated islet with a bimodal distribution in GK activity and a region of high frequency cells. B). Schematic of phase lag across simulated islet with a bimodal distribution in GK activity and a region of high frequency cells. C). Change in frequency of islet with indicated populations removed with respect to control islet with all cells present comparing bimodal model with a region of high frequency cells to a bimodal model with randomly distributed high frequency cells as in Fig 6. D). Change in frequency when high frequency region is removed compared to average oscillation frequency of remaining cells that indicates the expected oscillation frequency. Error bars represent mean ± s.e.m. Student’s t-test was performed for C and D (paired test) to test for significance. Significance values: ns indicates not significant (p>.05), * indicates significant difference (p < .05), ** indicates significant difference (p < .01), *** indicates significant difference (p < .001), **** indicates significant difference (p < .0001). Data representative of 5 simulations with differing random number seeds. (TIF) [file pcbi.1008948.s009.tif]

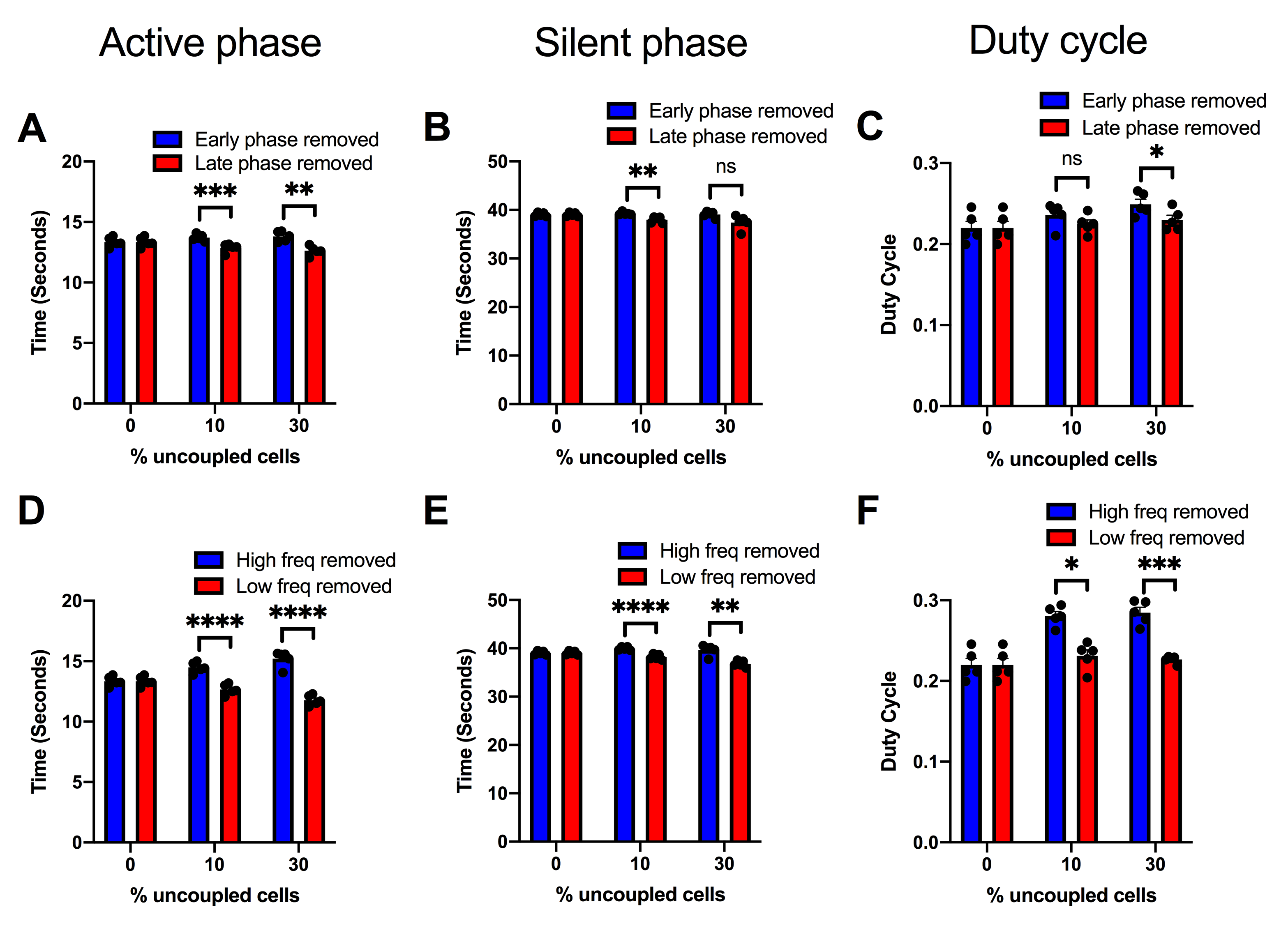

Supplement: S10 Fig — A). Change in mean duration of active phase when top 1%, 10% or 30% early/late phase cells are removed from simulations in Fig 4. B). Change in mean duration of silent phase when top 1%, 10% or 30% early/late phase cells are removed from simulations in Fig 4. C). Change in mean duty cycle when top 1%, 10% or 30% early/late phase cells are removed from simulations in 4. D). As in A for simulations when high/low frequency cells are removed from Fig 5. E). As in B for simulations when high/low frequency cells are removed from Fig 5. F). As in C for simulations when high/low frequency cells are removed from Fig 5. Error bars are mean ± s.e.m. Paired Student’s t-test was used to test for significance. Significance values: ns indicates not significant (p>.05), * indicates significant difference (p < .05), ** indicates significant difference (p < .01), *** indicates significant difference (p < .001), **** indicates significant difference (p < .0001). Data representative of 5 simulations with differing random number seeds. (TIF) [file pcbi.1008948.s010.tif]
