# Supplementary material for: Small subpopulations of β-cells do not drive islet oscillatory [Ca2+] dynamics via gap junction communication
Source: PLoS Comput Biol. 2021 May 3;17(5):e1008948. doi: 10.1371/journal.pcbi.1008948 (PMC8118513; doi:10.1371/journal.pcbi.1008948)
Supplement: S1 Table — Table describes the parameters in the computational model that are heterogenous for each cell. The mean and standard deviation is defined in the table. Changes to these parameter distributions are discussed in the methods. (PDF) [file pcbi.1008948.s011.pdf]

Parameter	Description of parameter	Mean	Standard Deviation	units
g_{KATP}	Max conductance of K_{ATP} channel current	2.31	0.57	pA mV^{-1}
g_{KTO}	Conductance of $I_{KCa(BK)}$ (voltage and Ca^{2+}) dependent transient outward K^+ current	2.13	0.213	pA mV^{-1}
P_{SERCA}	Maximum rate of pumping Ca^{2+} into ER	0.096	0.0096	amole ms^{-1}
P_{NaCa}	Maximum ampliture of I_{NaCa}, Na^+/Ca^{2+} exchanger	204	20	pA
P_{rel}	Converting factor for Ca^{2+} release from ER	0.46	0.046	fl ms^{-1}
P_{op}	Maximum rate of ATP production from oxphos	0.0005	0.00005	ms^{-1}
$[ATP_{tot}]$	Total amount of ATP species	4	0.4	mM
k_{glc}	Rate constant of glycolysys	0.000126	0.0000315	ms^{-1}
