# Supplementary material for: Small subpopulations of β-cells do not drive islet oscillatory [Ca2+] dynamics via gap junction communication
Source: PLoS Comput Biol. 2021 May 3;17(5):e1008948. doi: 10.1371/journal.pcbi.1008948 (PMC8118513; doi:10.1371/journal.pcbi.1008948)
Supplement: S2 Table — Table describes the parameters that have heterogeneous populations in computational model. The mean of each population is determined from the mean parameter value from unimodal normal simulations (See methods). (PDF) [file pcbi.1008948.s012.pdf]

Parameter	Description of parameter	Mean ‘early phase’ cell population	Mean ‘non-early phase’ cell population,	units
g_{KATP}	Max conductance of K_{ATP} channel current	2.3517	2.2930	pA mV^{-1}
g_{KTO}	Conductance of $I_{KCa(BK)}$ (voltage and Ca^{2+}) dependent transient outward K^+ current	2.12521	2.12793	pA mV^{-1}
P_{SERCA}	Maximum rate of pumping Ca^{2+} into ER	0.09666	0.09586	amole ms^{-1}
P_{NaCa}	Maximum ampliture of I_{NaCa}, Na^+/Ca^{2+} exchanger	204.98	203.84	pA
P_{rel}	Converting factor for Ca^{2+} release from ER	0.4548	0.4609	fl ms^{-1}
P_{op}	Maximum rate of ATP production from oxphos	0.00049742	0.00049957	ms^{-1}
$[ATP_{tot}]$	Total amount of ATP species	3.99813	4.00341	mM
k_{glc}	Rate constant of glycolysys	0.0001064	0.0001286	ms^{-1}
