# Supplementary material for: Small subpopulations of β-cells do not drive islet oscillatory [Ca2+] dynamics via gap junction communication
Source: PLoS Comput Biol. 2021 May 3;17(5):e1008948. doi: 10.1371/journal.pcbi.1008948 (PMC8118513; doi:10.1371/journal.pcbi.1008948)
Supplement: S1 Files — Files include those used to generate data in Figs 1, 2, 4 and 6. (ZIP) [file pcbi.1008948.s013.zip › Note.docx]

Model code for respective files
