# Supplementary material for: Small subpopulations of β-cells do not drive islet oscillatory [Ca2+] dynamics via gap junction communication
Source: PLoS Comput Biol. 2021 May 3;17(5):e1008948. doi: 10.1371/journal.pcbi.1008948 (PMC8118513; doi:10.1371/journal.pcbi.1008948)
Supplement: Attachment — Submitted filename: plos_ref_repPCOMPBIOL-D-20-01679.pdf [file pcbi.1008948.s014.pdf]

Reviewer:

Manuscript Number PCOMPBIOL-D-20-01679

"Small subpopulations of β-cells do not drive islet oscillatory [Ca2+] dynamics via gap junction communication

In their paper, Dwulet et al. investigate the effect of beta-cells heterogeneity on islet calcium oscillations through mathematical modelling and simulations. At first, they describe beta-cells physiology and recent findings on a subtype of beta-cells with increased metabolic activity, called "hub" cells. On this basis, they perform an extensive numerical study to quantify how cells heterogeneity, opportunely introduced to model beta-cells subtypes, affect calcium response, and to address whether such hub cells are required to drive islet calcium oscillations. Specifically, they simulate several islets composed by a heterogeneous population of cells and a heterogeneous distribution of gap junction conductance. They compute islet calcium oscillations in different conditions and quantify variations in calcium response when different types of cells are hyperpolarized or decoupled, such as the metabolically more active cells or cells with higher (or lower) oscillation frequency. Based on these simulations, they conclude that a specialized type of cells is unlikely to drive islet calcium dynamics as recent experimental investigations suggested it.

The paper is well written and address an important point in beta-cells and islet functioning. However, although the topic is of great interest, I'm failing to see "exceptional significance" that would be required from PLOS Computational Biology. In my opinion, the paper would fit within PLOS One after addressing the points, listed below.

1) The authors adopted the Cha-Noma model, which, if it appears to be very detailed in the electrical compartment, it seems to be more simplistic in the glycolytic component respect to other models in the literature. What can add the use of more detailed models? The authors should comment on this.

2) There is evidence that gap junction coupling may also allow metabolites diffusion. Indeed, some modelling scenarios included this aspect in their mathematical descriptions:
"Tsaneva-Atanasova, K., Zimliki, C. L., Bertram, R., & Sherman, A. (2006). Diffusion of calcium and metabolites in pancreatic islets: killing oscillations with a pitchfork. Biophysical Journal, 90(10), 3434-3446."
"Loppini, A., Braun, M., Filippi, S., & Pedersen, M. G. (2015). Mathematical modelling of gap junction coupling and electrical activity in human β-cells. Physical biology, 12(6), 066002."
To what extent this ingredient may affect simulations results? Authors should discuss this point.

3) It is not clear to me what is the exact size of the modelled islets. Also, it would be interesting to check if the emergent properties are size-dependent.

4) It is interesting to test for a bimodal distribution of gap junction coupling strengths. However, this modelling choice needs to be related to experimental observations suggesting this evidence, also if they come from other connexins type, or cells, such as in:
"Moreno, A. P., Eghbali, B., & Spray, D. C. (1991). Connexin32 gap junction channels in stably transfected cells: unitary conductance. Biophysical Journal, 60(5), 1254-1266."

5) It is well accepted that noise has significant effects on beta-cells emergent activity. Perhaps, it would be interesting to check if the combination of noise and heterogeneity would affect simulations results, as performed in other computational studies analyzing the effect of heterogeneity in small beta-cells aggregates:
"Loppini, A., Pedersen, M. G., Braun, M., & Filippi, S. (2017). Gap-junction coupling and ATP-sensitive potassium channels in human β-cell clusters: Effects on emergent dynamics. *Physical Review E*, *96*(3), 032403."

6) To check if the model is correctly reproducing the emergent dynamics of the islet as observed in experiments, the Authors should check if the highly metabolic cells or intrinsically faster cells correspond to functional hubs, i.e., cells with higher pair-wise correlation indices compared to the rest. This can be performed by a functional network analysis as performed in other studies:
"Stožer, A., Gosak, M., Dolenšek, J., Perc, M., Marhl, M., Rupnik, M. S., & Korošak, D. (2013). Functional connectivity in islets of Langerhans from mouse pancreas tissue slices. *PLoS Comput Biol*, *9*(2), e1002923."
