# Supplementary material for: Small subpopulations of β-cells do not drive islet oscillatory [Ca2+] dynamics via gap junction communication
Source: PLoS Comput Biol. 2021 May 3;17(5):e1008948. doi: 10.1371/journal.pcbi.1008948 (PMC8118513; doi:10.1371/journal.pcbi.1008948)

Response to Reviewers.

We thank the editor and reviewers for their efforts and for their thoughtful comments**.** Below follows our detailed response to each of the reviewers' comments (reproduced in *italics*), together with a description of where and how we have modified the manuscript. Where relevant we include data from new experiments. Changes in the manuscript are identified by underlined red. All line numbers are from the final red-underlined 'Markup' version.

Peer Reviewer #1

This is a very important and timely paper that appears carefully executed, I am favorably impressed. The paper uses a network model of the pancreatic islet to argue, in a quantitative way, that islet subpopulations cannot exert strong control over calcium dynamics via gap junctions. This issue was raised by Rorsman, Satin, and Rutter in two recent and highly charged Perspectives in Diabetes, and this paper is exciting because it provides a quantitative platform for (and against) their arguments. The model seems generalizable and robust, accommodating a range of biological possibilities with the same general conclusions.

We thank the reviewer for their enthusiasm about the manuscript and address their concerns below.

Major

1. The authors show that hyperpolarizing 10% of cells with high GK flux suppressed islet calcium, whereas hyperpolarizing cells with normal metabolic activity had no effect. This can only occur if there is a physiological relationship between high glucose oxidation and higher coupling to neighbors, particularly as removing these highly metabolic cells altogether did not suppress calcium. It may not be the metabolism of these cells that matters, per se, but the coupling. What is the experimental justification for linking high GK activity (directly or inversely) to coupling conductance? Is there any? Or is this an emergent property of the network?

We agree with the reviewer that the suppression of the islet by GK high cells may be due to an increase in gap junction coupling in these cells. A key part of this study (e.g. figure 3) was to investigate whether an increase in gap junction coupling in these GK high cells was required to impact whole islet dynamics. The experimental justification of linking high GK activity with high gap junction conductance was from Johnston et al., Cell Metabolism, 2016 in which a population of 'hub' beta cells with higher GK protein expression was identified. This population was demonstrated to show higher connectivity (correlated with more cells or had more links within the islet than 'non-hubs.') which was assumed to be due to higher gap junction coupling. As gap junctions are the known to coordinate calcium activity, this is an understandable assumption. However, as the reviewer is suggesting, this assumption is not valid. Increasing gap junction conductance in GK high cells does not allow these cells to exhibit greater control over the islet as they are in fact hyper-polarized by GK low cells. Further, we have included additional network analysis (see also response to reviewer 3) which demonstrates that GK high cells have increased links under conditions where gap junction conductance is not different from the rest of the islet (Fig.1E,J; Fig.2E). As such we conclude that GK high cells showing increased connectivity is an emergent property of the network, not a reflection of higher coupling conductance.

To address this comment, we have edited the results section to better describe the justification for introducing a correlation between GK activity and gap junction conductance, and added more discussion around these results and their implications – see Results line 251 and Fig.3 and S3 Fig; and Discussion lines 596-600 607-608. We have also included link analysis and describe these Results and Discuss them – See Fig.1E,J and Results lines 136-139; Fig 2E and Results lines 204-205, 229-230; Fig 4J and Results line 351-353.

2. Related to Results, Lines 264-266: "These cells [cells that fire before the rest of the islet] have been suggested to have higher intrinsic oscillation frequency [29, 40], which may lend themselves to act as rhythmic pacemakers to drive [Ca2+] oscillations across the islet."

a) *A minor point is that Reference 40 does not appear to addresses the relationship between frequency and pacemaking.*

This was an oversight and should have been reference 38 Salem et al. and has been fixed - see line 300.

b) *It seems wholly speculative that first responder cells would, on their own, possess higher frequency (as suggested in Line 274). If there is experimental evidence for this, please provide it.*

We previously investigated cells with earlier phase compared with the islet average in Westacott et al., Biophys J., 2017 and these cells at the 'wave origin,' as they were previously referred to, were found to have decreased NAD(P)H or metabolic activity. NAD(P)H was also found to be negatively correlated with frequency in uncoupled Cx36(-/-) islets. We therefore infered that cells that lead the calcium wave or have an earlier phase would have higher frequency and our simulations did confirm this (Fig 4). This had been updated – see Introduction line 98-99.

c) *Most importantly, why would high frequency cells lend themselves to pacemaking when increasing GK activity lowers oscillation frequency (as stated in Lines 272-274)?*

There are two justifications for this: 1) Firstly, in another study Salem et al., Nature Metabolism, 2019 examined cells that lead the oscillations (ie show earlier, lower phase oscillations) which they referred to as 'leader cells.' When leader cells were experimentally photo-ablated, they were able to disrupt Ca^{2+} activity across the islet, suggesting these cells may act as pacemakers. 2) Secondly in our previous study, Westacott et al., Biophys. J., 2017, cells that lead the oscillations (ie show earlier, lower phase oscillations) also have higher intrinsic oscillation frequencies and lower metabolic activity. This study also demonstrated that within a generalized coupled oscillator model higher frequency cells lead the oscillations (ie show earlier, lower phase oscillations). Thus, akin to cardiomyocytes, would such higher frequency cells act as a rhythmic pacemaker? These studies both motivated us to test whether cells with earlier, lower phase oscillations did have higher frequency and if they could act as pacemakers; either driving the oscillation frequency or maintaining oscillations. However, our results may confirm the reviewer's hesitation in thinking that higher frequency cells are pacemakers. Our results suggests that small populations of cells with higher frequency and lower GK activity are not pacing the islet. In fact, the later phase cells with lower frequency and high GK activity had a greater effect on the frequency of the islet and lowered the overall islet frequency (Fig 4-5).

To address this topic, we elaborate on this motivation – See Introduction lines 96-99.

Minor

1. As written, the short title isn't accurate and should be modified – even single cells can generate calcium oscillations.

We agree this short title was confusing and have revised it to "Small β-cell populations cannot drive islet-wide Ca^{2+} dynamics" (line 16).

2. While Lines 90-101 of the introduction do a nice job of introducing the field, I'm not thrilled with the abstract. Don't get me wrong, I greatly appreciate the neutral tone taken by the authors, however they have divorced their findings from the experiments that motivate this paper, and therefore undersell the papers significance:

a) There is an important experimental rationale for these modeling studies that is told in introduction but not the abstract. The authors did not choose these specific manipulations of

subpopulations at random – can the backstory that motivates this paper be somehow acknowledged in the abstract to enhance the impact?

b) The authors run simulations with both removal and hyperpolarization of highly metabolic cells. It could be clarified that hyperpolarization mimic optogenetic silencing. What experiment does removal mimic, laser ablation?

c) Abstract, lines 29-31: It is not clear what is meant by "increased excitability". It is also not clear whether the authors refer to slow metabolic or fast electrical oscillations, which interact and both contribute to excitability.

d) Abstract, line 32: "metabolic activity" should be referred to more specifically as glucose oxidation (assuming the authors mean changes in GK flux).

We agree with the reviewer and thank them for their detailed critique that the abstract is lacking in displaying the importance of the experiments that motivate the paper, which is important in reinforcing why the results are significant and impactful in the field. The abstract now includes the experiments that motivate the paper and how we translated these experiments to simulations, including clarifying that hyperpolarization mimics optogenetic silencing and removal of populations mimics laser ablation (lines 26-33). We also clarified wording by removing excitability (line 39) because it was unclear that we focused on metabolic activity of beta cells in this manuscript. We also clarified that metabolic activity does refer to GK flux and now states that we investigated metabolic activity through glucose oxidation (line 33).

3. Introduction, line 94: 10% of beta cells are highly excitable… and had higher metabolic activity [29]. Excitable at lower glucose (recruitable?) Higher NADH response to glucose?

We reworded this sentence to explain that here excitable means that they are able to recruit calcium in many other cells at low glucose – See Introduction lines 91-92.

4. Results, Lines 112-121: The first paragraph of the Results in largely redundant with the introduction.

We agree with the reviewer and have removed several sentences in the second half of the first paragraph of the results and adjusted the last sentence – See Results line 116-118.

5. Lines 76-77: References 9-11 do not do justice to the statement that "disruptions to this GSIS pathway occur in diabetes".

We agree that these references do not display just how many parts of this pathway are affected in diabetes. We have added several additional references that include disruptions to K_{ATP} channels, GK, exocytosis and insulin release that occur in diabetes pathogenesis – See Introduction line 75.

6. Line 508: "Duty cycle is a large determinant of insulin release, thus a ~10% reduction would not be expected to impact insulin release substantially." This statement is misleading and almost certainly false.

We agree this is perhaps a misstatement, given that the precise link between duty cycle and insulin release is not firmly established and is very likely to not be linear. Thus while a 10% reduction is relatively minor in impacting duty cycle, we cannot assume this translates to a minor disruption to insulin release. We do note that since we have removed the 10% cells with highest metabolic activity some reduction in duty cycle would be expected. We have also investigated a new skewed distribution (see reviewer 2 comment) and in this case the reduction in duty cycle is closer to a 40% reduction which is significant, although not a near-complete reduction as experimental evidence

suggests. However in this latter case the effect of targeted hyperpolarization shows the least agreement with prior experimental evidence.

To address this comment we describe these newer results – See Results lines 228-229. We also discuss the link between duty cycle and insulin release, avoiding the misleading statement previously included – See Discussion lines 547-556, 559-560.

7. Line 593: "This also suggests that slow metabolic oscillations will better coordinate [Ca2+] dynamics across the islet, rather than purely a faster-oscillating electrical subsystem." Haven't prior modeling studies by Sherman and colleagues already shown this? If so they should be cited in the Discussion.

We agree with the reviewer that the work by Sherman and colleagues should have been cited to support this point. Sherman and colleagues suggests that there are 3 ways to achieve medium bursting across the islet, accounting for combinations of fast and slow oscillators (Zimliki et al., Biopyhys J., 2004). Here, we suggests that the slow oscillators are more important for coordinating the activity of the islet. This reference and discussion has been added – see Discussion lines 653-655.

Peer Reviewer #2

For complex systems such as pancreatic islets, computational simulations such as those presented here are crucial tools for testing and generating hypotheses. The focus of this manuscript, whether specialized subpopulations can control islet activity, is important and timely given recent developments and significant ongoing debate in the field. However, I have some concerns relating to methods used to generate parameter sets for the computational islets in the study. The cases of parameter bimodality and correlation seem unrealistic, and some of the main results seem to reflect artefacts in how these parameter distributions were generated. The conclusions drawn by the authors are supported by the results presented, but they are tightly tied to the parameter choices. I believe these parameter distributions could be improved in a straightforward way, but this would involve potentially significant changes to the results and possibly conclusions of the paper. Therefore, in its current form I cannot recommend the manuscript for publication.

We appreciate the reviewer's positive comments regarding this manuscript being important and timely. We agree that the parameters distributions could be greatly improved upon, and we appreciate the reviewers thoughtful comments towards this significant point. In response we have made significant changes that are addressed further below. The results from these new distributions did not significantly change the conclusions in the paper and this argues against the conclusions being tightly tied to parameter choice. Thus we argue that our results do provide significant insights into intact islet dynamics that may be used to guide future research.

Major comments

The method for generating correlated parameter distributions gives a highly artificial distribution of parameter combinations, which is the direct cause of the results observed in those simulations. A more realistic method will give 2D random variables, which as a scatter-plot will be a cloud of points in Kglc-gcoup parameter space with a specified correlation. This is easily achieved given the two marginal distributions for GK (Normal) and gcoup (gamma) using a copula. See how to do this in Matlab here: https://www.mathworks.com/help/stats/copulas-generate-correlated-samples.html
I also attached an example figure and Matlab code showing the result I'm imagining instead of the values shown in FigS2A. A similar approach should be used for other simulations with correlated parameter distributions (e.g., Fig3, FigS2B). Note that the same code easily generates negatively

correlated (rho<0) or uncorrelated (rho=0) distributions. I suspect this will give more interesting results - there will be a more natural distribution of GK and gcoup parameter values across the islet.

We want to thank the reviewer for this excellent suggestion and agree that the original 2D distributions were unrealistic. We have implemented the copula to make the correlation between k_{glc} and g_{Coup} more realistic in Fig 3 (see panel A below). The results here indicate that this 2D distribution left-shifts the ability of the cells to suppress activity, allowing fewer cells to suppress [Ca^{2+}] across the islet upon hyper-polarization. However, introducing the correlation between k_{glc} and g_{Coup} leads to reduced difference between GK^{High} and GK^{Low} cells (see panel B below), thus deviating from experimental data.

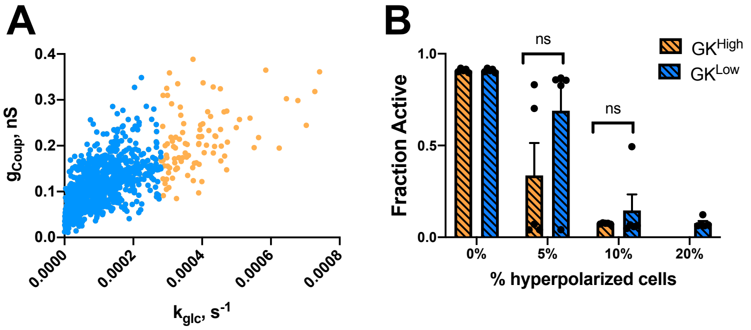

This same approach was also used to correlate g_{Coup} and k_{glc} for the continuous model (S3 Fig, below) and also inversely correlate g_{KATP} (S3 Fig). Here, the revised correlation provides a small left shift, but does not affect the difference between GK^{Higher} and GK^{Lower} cells.

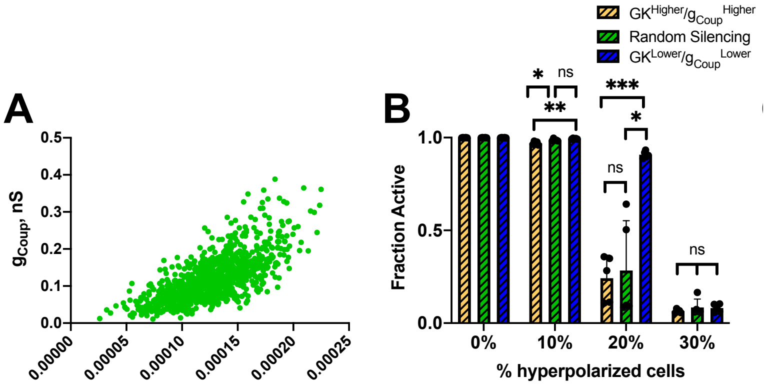

In response to this comment we have modified <u>Fig. 3 and S3 Fig</u>. to include data including this refined 2D distribution. We also describe this data – <u>see Results lines 255-270, Fig 3 caption lines 272-280, and lines 182-185, 187-188</u>. We also add additional discussion related to these results – <u>see Discussion lines 596-600, 607-608</u>. We also describe the methodology to generate these distributions – <u>see Methods lines 758-762</u>.

Another concern regarding the selected parameter distributions is the sharp, highly separated bimodal distributions: these seem unrealistic. Furthermore, the constraint of keeping the global distribution's mean constant seems to introduce undesired artefacts. For example, in the bimodal kglc case, lowering the mean kglc of GK-low cells, by construction, makes the islet more susceptible to silencing than the default (Fig1) case, because 90% of cells have lower kglc. Similarly, reducing the mean gcoup in the low-gcoup group, the islet is less coupled overall and thus less sensitive to interventions done on the GK-high/high-gcoup group. Is such a sharp bimodal distribution desired and/or realistic? An alternative is to compare a right-skewed distribution for Kglc to the Normal distribution case (perhaps gamma, or some distribution motivated by data on GK activity levels if available)

We again agree with the reviewer regarding their concern with the bimodal distribution and thank them for their suggestion. We have implemented an alternative model where we applied a

gamma distribution to k_{glc} and refer to this as a unimodal skewed distribution in the manuscript (See A below). This distribution was defined to have the following constraints: The top 10% of cells with the highest GK activity have an average of 3X the overall islet average k_{glc}. We imposed this assumption because in Johnston et al., Metabolism, 2016, found that the protein levels of GK in the hub cell population had ~3X the average GK. We do think this distribution is more realistic and has a much broader variation (which addresses another concern by the reviewer). With this new skewed distribution the GK^{High} cells are able to suppress the activity of the islet when all (10%) GK^{High} cells are hyperpolarized (See B, below). The difference in suppression between GK^{High} and GK^{Low} cells is lessened by this broader distribution as compared with the original bimodal distribution, and thus is less descriptive of experimental data (Johnston et al. Cell Metabolism 2016). As such, we retain the bimodal distribution in the manuscript to show the differences between these two distributions on the effect of hyper-polarization.

To address this comment, we now include results for this skewed to Fig 2, Fig 3 and S1 Fig and updated captions lines 210-221, 272-280, and 1028-1030 respectively. The description of results has been adjusted to account for skewed and bimodal distribution – see Results lines 193-202 (skewed distribution) and lines 233-245 (bimodal distribution). The methods have been updated to include a description of this distribution and an updated description of the bimodal distribution – see Methods lines 740-756.

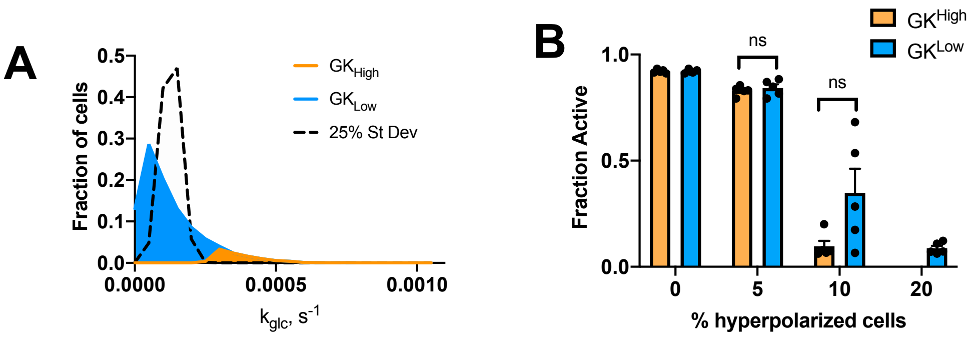

The parameter sets shown in the figure seem to have very tight distributions. By comparison to Fig1E/FigS1A, the distributions for Kglc in Figures Fig2B, Fig3A/D, and FigS1B-D all appear to show standard deviation of 1% of the mean, but the text claims simulations were done with 25%. The exception is Fig2A, which appears as the stated 25% deviation for Kglc. – this should be stated somewhere.

We agree with the reviewer that this information should have been included and is actually at 2.5% for the bimodal distribution, a 10% reduction in standard deviation. To address this comment, we include now this information - see Results line 235 and methods line 755.

Other comments

Hyperpolarization vs decoupling are different conditions, which could be made clear early in the manuscript instead of just in the discussion. Hyperpolarization of a cell (or cell subpopulation) will generate inhibitory currents in neighbor cells via gap junctions, while decoupling does not. This could be used as the guiding principle for explaining the results of the paper.

This is an excellent suggestion that was also suggested by another reviewer and should be clearer in the paper since the results greatly depend on which method is used. We have added to the abstract to explain that we are using these methods to mimic experimental findings that motivate the paper - see Abstract line 30-33. We have also clarified this in the results when we first introduce the methods – see Results line 122-124.

Terminology suggestion for parameter distributions: "unimodal" instead of "continuous" for parameter distributions. Terminology suggestion for phase analysis: early/late phase.

We agree that the terminology could be clearer with some changes. We changed the terminology of the “continuous” distribution to “unimodal normal” distribution throughout. We also added a “unimodal skewed” distribution to address concerns listed above about the bimodal distribution being unrealistic and since they are both unimodal, we clarified the difference with the word normal or skewed. We also changed all “low/high phase” to “early/late phase” throughout the paper and in the figures (Figs. 4, 5 S5, S7, S10).

For all bar-charts, the data points per simulation could also be shown to give an idea of the variability (as was done in FigS5)

We added all data points to show variability between simulations. See all figures and supporting figures.

From the simulation code, it appears that variation in several cell parameters was also included (e.g, gKATP). This is also implied in the description of modeling low/high phase cells. It would be helpful to list these parameters with heterogeneity clearly in the first section of Methods, even if they are the same as prior modeling studies with the same model.

We also added a table, now Table S1 describing the parameter heterogeneity for the model before changes were made to account for alternative distributions. We now refer to this table in the methods. Table S2 now describes the parameter heterogeneity when changes were made in the bimodal frequency population presented in figure 6. See Table S1, Methods line 709-711 and figure caption line 1157-1159. .

The text does not clearly indicate which value is used for computation of Icoup in a given cell - the value of g_coup^(i,j) isn't defined. In the code for the simulations, it is clear that the average value of gcoup from cell i and j is used to compute Icoup. This was described in previous manuscripts using this model (eg. Westacott et al 2017), and it would be helpful describe it in the methods section here as well.

This was previously described further under the section ‘modelling changes in coupling’, but we moved it earlier to the description of the model to make it clear how we calculate I_{Coup} and how g_{Coup} is distributed as a gamma distribution with an average of g_{Coup} = 120pS. See Methods lines 707-709.

The average gcoup is claimed to be ~120pS in the text, but Fig3A/D, FigS1, and FigS2A shows ~0.12pS. Should the yaxis show nS instead of pS?

This was an oversight, where gcoup should be expressed as 120pS or 0.12nS. This has been corrected in all places – See Fig.3, S1 Fig, S2 Fig.

Why is ghyper = ghyper'(1-p0_KATP) when hyperpolarization is on? Shouldn't this be an ohmic current independent of KATP channel open probability, for example as a simple model of a photo-activated chloride current?*

This is a good point. The study in Johnston et al utilized eNpHr3.0 photoactivation which is indeed a chloride current. However we reasoned a simple ohmic Cl- current that is photoactivated

would be unsuitable: given the high Cl- concentration within the beta cell, the reversal potential is relatively high, such that under some conditions a Cl- current can be stimulatory. In contrast eNpHr3.0 shows light-driven Cl- influx that generates a strong positive current: it is a light driven Cl- pump rather than a light-gated Cl- channel. Thus, to reproduce such a current we chose an ohmic current similar to a K+ leak current that is photoactivated. p0 is included to maintain stability of the model – we note p0_KATP is <<1 such it has little practical impact. However its inclusion originally was to avoid model instability when KATP heterogeneity was exaggerated. We did slightly change the model to show this should be equivalent to an ohmic current - see line 736.

It seems that the low/early-phase cells clustered in the same region probably because the simulation is deterministic. If noise is introduced (e.g., randomized initial conditions; a noise current in the voltage equation for each cell) does the location of low-phase change (possibly from burst to burst)? If there are sufficiently many low-phase cells with very similar intrinsic frequencies, it seems this could be possible, but if there is a clearly dominant "lowest-phase" region, even noise may not be able to shift the burst-initiation site.

This is an interesting suggestion that the wave may start at different regions in the islet over different oscillations. We tested this by adding noise to our simulations (in K_{ATP} current as we have previously performed – Notary PLoS Computational Biology 2016) and investigated whether the top 10% of early phase and late phase cells changed from oscillation to oscillation. Although only ~70% early phase (C below) and ~60% late phase (D) cells remained stable from oscillation to oscillation, the region within the islet that the wave begins in started in the same approximate location for all 5 seeds over 3 oscillations except 1 oscillation for 1 seed (shown in B oscillation 3).

The shift in differences in % overlap for early and late phase cells from oscillation to oscillation is most likely due the fact that the 'middle phase' cells respond at nearly identical times and are therefore more difficult to differentiate. Since the starting region of the calcium wave did not change from oscillation to oscillation for all but 1 oscillation in one seed, we did not include this in our analysis. A and B show the least consistent seeds of those examined. A has 84% consistency for oscillation 1 and 2, 80% consistency between oscillation 1 and 3 and 66% consistency between oscillation 1 and 3. However it is clear that the wave still starts in the same region across all oscillations. For B there is 94% consistency between oscillation 1 and 2, but only ~23% consistency between oscillation 3 compared to both oscillation 1 and 2.

We do not include this data within the manuscript to avoid breaking the flow. However if the reviewer believes it critical we can make it available.

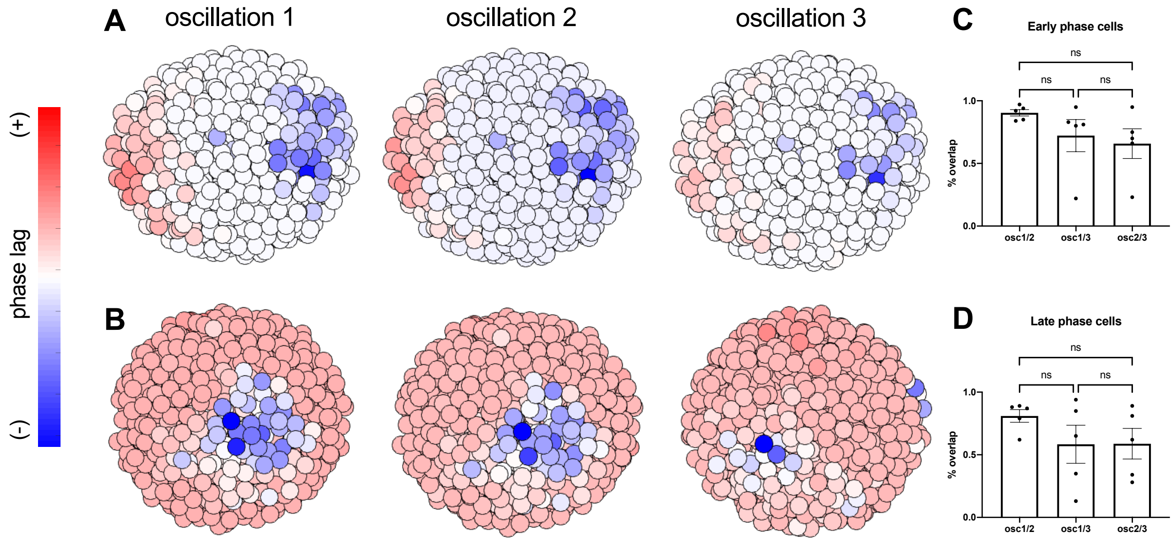

The coupling currents are defined by the voltage difference between cells, so whether the current is inward or outward is defined by which cell is leading vs lagging - gap junctions will try to bring the two voltages towards each other. Perhaps a more informative analysis than characterization of whether subpopulations have inward/outward currents during different phases of a burst would be to consider the value of gcoup relative to the cell's total conductance. A more significant effect of coupling will occur when the coupling conductance is relatively large compared to the cell's total conductance at that moment. This would be particularly important for cells at rest with low total conductance in that state.

Again, we want to thank the reviewer for this suggestion. We examined the time-course for the total value of g_{Coup} relative to the cell's total membrane conductance and found that it has a similar relationship to the current relationship. During the upstroke, the magnitude of the relative conductance ($g_{membrane}$/ g_{Coup}) is highest, and somewhat less at the end of the oscillation. However elsewhere the magnitude of the relative conductance is very low - see A for calcium trace vs B relative conductance below.

We do note the reviewers expectation for this is reasonable, and was our initial expectation. Indeed on some occasions where there is a large time difference in the upstroke between neighboring cells there is a brief spike in Icoup - see Figure 7 C second pulse for such an example. However outside of these cases the difference in the regulation of membrane depolarization leads to a more sustained gap junction-mediated current

As such, coupling conductance has a greater impact during the established active phase where membrane conductance is relatively low; and to a much lesser degree during the initiation of the active phase where membrane conductance is relatively high. This matches our conclusions in the paper that coupling conductance is most important during the established active phase, rather than the upstroke or downstroke.

A

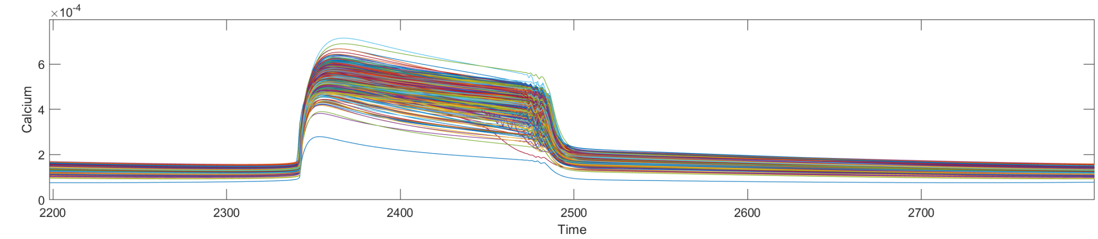

B

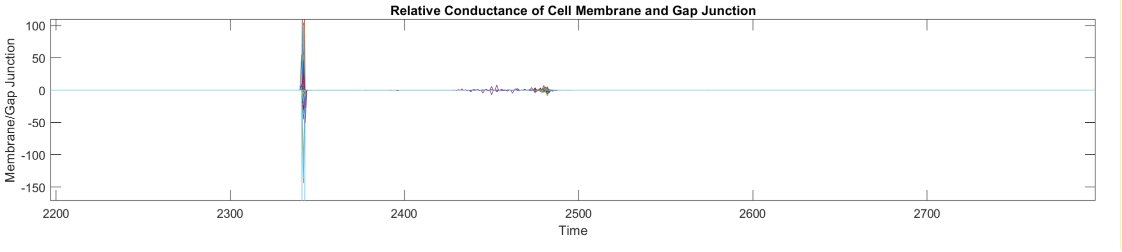

Peer Reviewer #3

In their paper, Dwulet et al. investigate the effect of beta-cells heterogeneity on islet calcium oscillations through mathematical modelling and simulations. At first, they describe beta-cells physiology and recent findings on a subtype of beta-cells with increased metabolic activity, called "hub" cells. On this basis, they perform an extensive numerical study to quantify how cells heterogeneity, opportunely introduced to model beta-cells subtypes, affect calcium response, and to address whether such hub cells are required to drive islet calcium oscillations. Specifically, they simulate several islets composed by a heterogeneous population of cells and a heterogeneous

distribution of gap junction conductance. They compute islet calcium oscillations in different conditions and quantify variations in calcium response when different types of cells are hyperpolarized or decoupled, such as the metabolically more active cells or cells with higher (or lower) oscillation frequency. Based on these simulations, they conclude that a specialized type of cells is unlikely to drive islet calcium dynamics as recent experimental investigations suggested it.
The paper is well written and address an important point in beta-cells and islet functioning. However, although the topic is of great interest, I'm failing to see "exceptional significance" that would be required from PLOS Computational Biology. In my opinion, the paper would fit within PLOS One after addressing the points, listed below.

We thank the reviewer for their time and appreciation of the manuscript, especially that it addresses an important point. We do wish to emphasize the significance of this manuscript, because we address a highly charged and controversial topic within the field of islet biology as to whether or not specialized beta cell subpopulations can drive the function of the whole islet. Results from our simulations indicate that two identified 'pacemaker like' populations cannot play a physiological role in driving islet function, at least under the mechanism proposed (with alternative mechanisms being purely speculative at this point). This is despite us being able to reproduce experiments from which these conclusions were made. We suggest that the experiments have natural artifacts in the way they are performed that do not allow the whole story of islet dynamics to be teased out. Given the significance of these findings we believe a forum such as PLoS Computational biology would be needed to communicate these results. Further is would be general interest in these studies as they address the important concept of cell heterogeneity and how it impacts tissue function.

1) The authors adopted the Cha-Noma model, which, if it appears to be very detailed in the electrical compartment, it seems to be more simplistic in the glycolytic component respect to other models in the literature. What can add the use of more detailed models? The authors should comment on this.

We agree that the glycolytic oscillator model is detailed and highly robust in describing metabolic and calcium oscillations. However, there has not been much exploration of metabolic oscillations in the context of a coupled islet model. Thus, how oscillations would coordinate between cells and how cells with differing metabolic oscillations would influence each other has not been explored significantly. Most importantly, the prior experimental studies which influence and motivate the study described here do not show metabolic oscillations or slow Ca^{2+} oscillations – see Salem et al Nature Metabolism 2019 or Johnston et al Cell Metabolism 2016. As such it would be overly speculative to incorporate metabolic oscillations into the model for this study. We do agree given their physiological importance, it would be interesting and important to investigate how subpopulations act within this model in the future. These points are now discussed – see Discussion lines 609-611, 618-620.

2) There is evidence that gap junction coupling may also allow metabolites diffusion. Indeed, some modelling scenarios included this aspect in their mathematical descriptions:
"Tsaneva-Atanasova, K., Zimliki, C. L., Bertram, R., & Sherman, A. (2006). Diffusion of calcium and metabolites in pancreatic islets: killing oscillations with a pitchfork. Biophysical Journal, 90(10), 3434-3446." "Loppini, A., Braun, M., Filippi, S., & Pedersen, M. G. (2015). Mathematical modelling of gap junction coupling and electrical activity in human β-cells. Physical biology, 12(6), 066002."
To what extent this ingredient may affect simulations results? Authors should discuss this point.

As the reviewer indicates, it has been suggested that gap junctions in the islet may allow metabolite diffusion and these modeling papers do consider the presence of metabolite diffusion. However there is not strong evidence that Cx36 gap junctions in the islet allow metabolite diffusion; rather several studies indicate the strong selectivity for cationic molecules, especially during stimulation of insulin secretion (Charpantier et al., Diabetologia, 2007, Bukauskas, J.Membr Biol, 2012, Harris, Prog Biophys Mol Biol, 2007). We are aware of a very recent paper in pre-print that

suggests metabolite diffusion occurs through gap junctions, Rao and Rizzo, BioRxiv, December 2020, however we do not believe the evidence presented in that study truly shows metabolite diffusion via gap junctions. Further we do note that the integrated oscillator model would suggest that the islet may coordinate with only electrical coupling (Bertram et al. Diabetes., 2018); although we do acknowledge this hasn't been investigated in depth. This was also suggested in one of the articles above (Tsaneva-Atanasova et al). We also do not consider slow metabolic and calcium oscillations in our study, as discussed further above, because these have not been investigated in the context of the subpopulations we study here. Thus any inclusion of metabolic coupling would be overly speculative. However we agree if metabolic coupling does occur, it could provide an alternative way that cells exhibit control across the islet and this would needs to be further investigated.

In response to this comment we include discussion of these points - <u>See Discussion lines 611-617</u>.

3) It is not clear to me what is the exact size of the modelled islets. Also, it would be interesting to check if the emergent properties are size-dependent.

We thank the reviewer for this suggestion. We investigated whether there is a size dependence affecting our results. The simulated islets in the manuscript are 1000 cells <u>(noted in the methods section lines 738 and 751</u>) but has also been added earlier in the methods when initially introducing the model (<u>line 709</u>). We also investigated smaller islets at 300 cells and larger islets at 2000 cells. When we hyperpolarized cells under 25% (A-C) and 50% (D-F) variation in GK (similar to Fig1) in smaller islets of 300 cells, the differences between hyperpolarizing cells with higher GK and lower GK were not significant as compared to a similar differences in larger (1000 cell and 2000 cell) islets. However, in any sized islet our conclusions that >20% of cells needed to fully suppress islet dynamics held true. We also investigated whether there was a size dependence that could affect the frequency of the islet. We performed simulations similar to Fig 4 in the manuscript and found that in different sized islets (G, I) we obtained very similar results to our original simulations of 1000 cells (H). The frequency of the islet did not change until at least 30% of early or late phase cells were uncoupled. Since the results between the 3 different sizes of islets were very similar, we did not include this data within the manuscript.

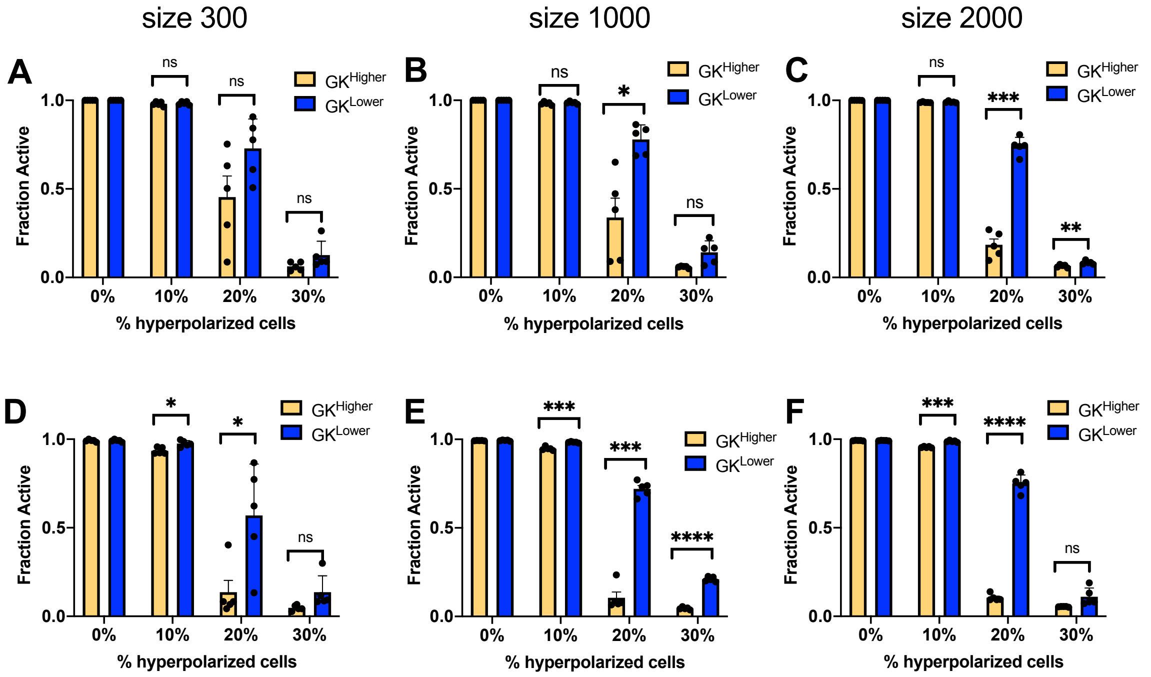

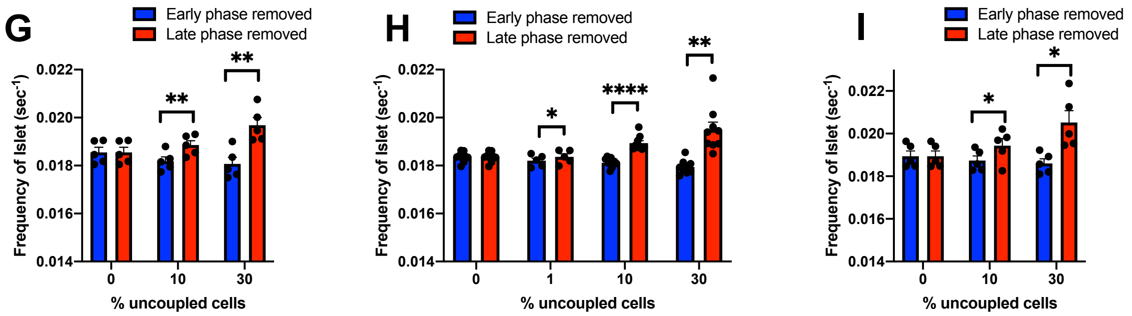

4) It is interesting to test for a bimodal distribution of gap junction coupling strengths. However, this modelling choice needs to be related to experimental observations suggesting this evidence, also if they come from other connexins type, or cells, such as in:
"Moreno, A. P., Eghbali, B., & Spray, D. C. (1991). Connexin32 gap junction channels in stably transfected cells: unitary conductance. Biophysical Journal, 60(5), 1254-1266."

We agree that this modelling choice should be based on experimental evidence. We originally performed these simulations to test what happens when the system is forced to an extreme circumstance. However we also note that the distribution of coupling conductance used in most cases in the manuscirpt is based on experimental evidence that found gap junction conductance to follow a skewed gamma distribution with a mean of 120pS (Farnworth et al., Journal of Physiology, 2014). Given other data that also does not show a bimodal distribution (e.g. Moreno et al., Endocrinology and Metab., 2005) we have removed this bimodal simulation of the coupling conductance. Instead we retain throughout the distribution that is based upon experimental evidence in situ. In addition, in response to reviewer 2 we also include other more realistic distributions of GK and correlate k_{glc} and g_{coup} more realistically – see Fig.2 and Fig.3. See also reviewer 2's comments for more discussion of these changes.

5) It is well accepted that noise has significant effects on beta-cells emergent activity. Perhaps, it would be interesting to check if the combination of noise and heterogeneity would affect simulations results, as performed in other computational studies analyzing the effect of heterogeneity in small beta-cells aggregates:
"Loppini, A., Pedersen, M. G., Braun, M., & Filippi, S. (2017). Gap-junction coupling and ATP-sensitive potassium channels in human β-cell clusters: Effects on emergent dynamics. Physical Review E, 96(3), 032403."

We thank the author for this suggestion and agree that noise can have significant effects. Therefore, we checked what effect noise would have on our results. We reran simulations for Fig 1 where GK rate had 25% variability (see A below) and 50% variability (B), and Fig 2 skewed bimodal distribution (C); each with noise incorporated into the K_{ATP} channel current (as utilized previously in Notary et al PLoS Computational Biology 2016 where noise had a significant impact). We did not find differences from our results: For a continuous distribution, at least 20% of hyperpolarized cells were still needed to completely silence the activity of the islet and for a skewed distribution in GK, at least 10% of cells were still needed for GK High cells to silence activity. We now include these data in the manuscript – see S2 Fig and figure caption on lines 1035-1044, Results lines 175-178 (25% and 50% variation) and lines 205-206 (bimodal distribution). We also included how we calculate noise – see Methods lines 793-799.

We also investigated the effect of noise on early phase cells and whether they remain the same cells from oscillation to oscillation under noise conditions. See reviewer 2 comments.

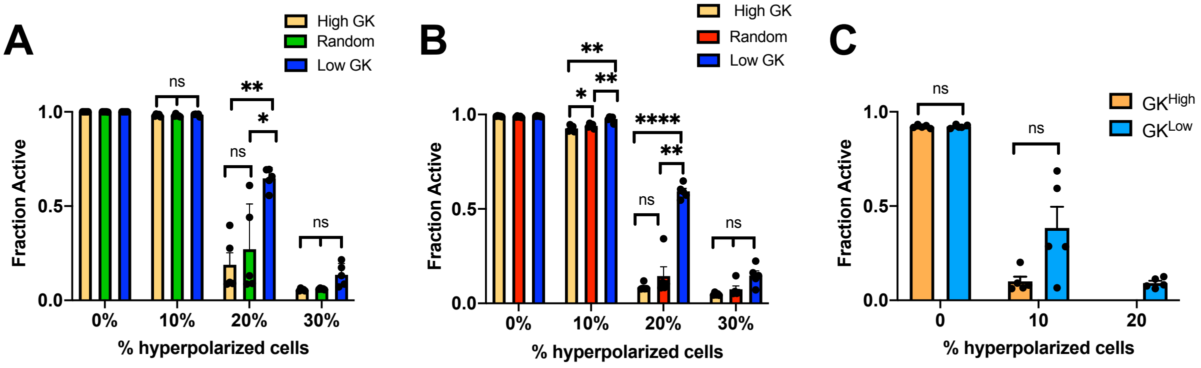

6) To check if the model is correctly reproducing the emergent dynamics of the islet as observed in experiments, the Authors should check if the highly metabolic cells or intrinsically faster cells correspond to functional hubs, i.e., cells with higher pair-wise correlation indices compared to the rest. This can be performed by a functional network analysis as performed in other studies: "Stožer, A., Gosak, M., Dolenšek, J., Perc, M., Marhl, M., Rupnik, M. S., & Korošak, D. (2013). Functional connectivity in islets of Langerhans from mouse pancreas tissue slices. PLoS Comput Biol, 9(2), e1002923."

We want to thank the reviewer for this excellent recommendation and agree this is important to add. We now have included a network analysis of links to compare links between high GK vs low GK cells or early/late phase cells. We have included this in Fig 1, 2, and 4.

For the continuous model at 25% (A below), 50% variation (B), and for a skewed distribution in GK (C), all cells with higher GK had more links than cells with lower GK. This means that these cells had calcium oscillations that were correlated with more cells within the islet. These results are in agreement with experimental data in Johnston et al Cell Metabolism 2016 in which hub cells that showed the highest number of links also showed increased GK expression. However we do not observe early phase cells to show an increase in the number of links, likely as they also do not show higher GK activity.

In response to this comment, we include results of this analysis related to high/low GK – see Fig 1 (caption on lines 147 and 151) with results on lines 136-139, and Fig 2 (caption on lines 216) with results on lines 204-205, 229-230. We include results of this analysis related to early/late phase – see Fig 4 (D) (caption line 325) and lines 351-353. See also Discussion lines 596-600 and Methods lines 782-791 under 'Network analysis of links'.

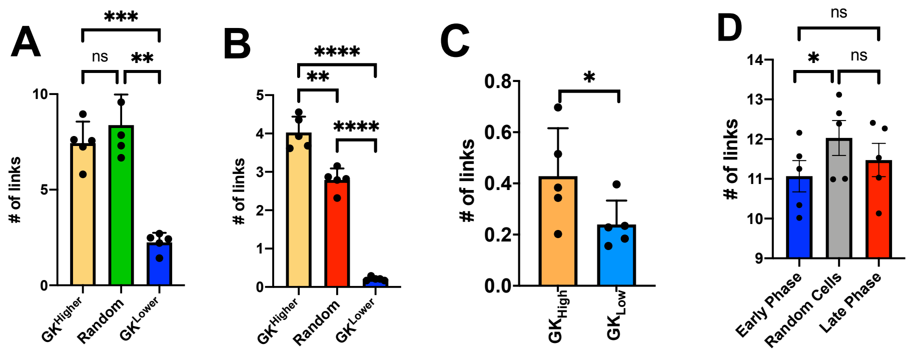

Supplement: Attachment — Submitted filename: Response to Reviewers 2 RB.pdf [file pcbi.1008948.s015.pdf]
