# Supplementary material for: Small subpopulations of β-cells do not drive islet oscillatory [Ca2+] dynamics via gap junction communication
Source: PLoS Comput Biol. 2021 May 3;17(5):e1008948. doi: 10.1371/journal.pcbi.1008948 (PMC8118513; doi:10.1371/journal.pcbi.1008948)
Supplement: Attachment — Submitted filename: Response to Reviewers 2nd v2.docx [file pcbi.1008948.s016.docx]

**Response to Reviewers.**

We thank the editor and reviewers again for their efforts in reviewing our revised manuscript. We thank them for the enthusiasm about the revised manuscript and for their final comments**.** Below follows our detailed response to each of the reviewers’ comments (reproduced in *italics*), together with a description of where and how we have modified the manuscript. Changes in the manuscript are identified by underlined red. All line numbers are from the final red-underlined ‘Markup’ version.

***Reviewer #1:*** *I have only two minor comments:*

We thank the reviewer for these additional comments and agree that the duty cycle comments could be improved upon. *1) Introduction, line 74: comma not semicolon.*

We have corrected this on line 74.

*2) Discussion, line 550: “Increased [Ca2+] duty cycle correlates with elevated glucose stimulation and insulin release [51, 52], thus a ~10% reduction would not be expected to impact insulin release substantially.”
 There is evidence to the contrary. A 10% change in duty cycle may well be meaningful for insulin secretion (PMID 7703919). Furthermore, the relationship between GK, duty cycle, and exocytosis is more complicated than the authors present. In their discussion the authors might should acknowledge that duty cycle and oscillation period, while initially set by GK, are then modified downstream by PK, which reduces duty cycle and period while at the same time increasing the steepness of the correlation between GK activity and exocytosis (PMID 33147484).
 This comment also applies to Lines 664/665 that discuss oscillation frequency.*

We thank the author for this insight and have added to the discussion that other downstream elements, such as pyruvate kinase, complicate the link between Ca^2+^ oscillation duty cycle and insulin secretion. The discussion includes the non-linear relationship of duty cycle to insulin secretion and how pyruvate kinase can increase oscillation frequency and decrease duty cycle while and amplifying insulin secretion. We also mention how pyruvate kinase increases Ca^2+^ oscillation frequency, differently to increased GK. See Discussion lines 554-562 and lines 647-652.

***Reviewer #2:*** *The revisions done by the authors have significantly improved the manuscript, and have clarified the conclusions of the study. The modeling has been done carefully, and represents well the current experimental knowledge of the relevant beta cell and islet properties. It is noteworthy that despite the changes (improvements) to the parameter distributions used in simulations, the main conclusions of the manuscript remain unchanged. This indicates that the results are robust to modeling choices, and strengthens the conclusions. All my major concerns have been addressed.*

We thank the reviewer and appreciate the enthusiasm for the revised manuscript. We again thank the reviewer for providing the comments about improvements to the parameter distributions. We believe this improved the manuscript greatly. *I have only a few more comments/suggestions that are largely cosmetic, which I leave to the authors to adopt or not based on their preference.

Line 106 - By "highly excitable" do you intend to refer to "highly metabolically active"? In beta cells, higher metabolic activity should lead to higher excitability (via KATP channels), but there could be non-metabolic reasons for higher/lower excitability. In any case, it seems useful here to specifically mention "highly metabolically active cells" as they (high GK cells) are a main focus of the manuscript.*

We agree with the reviewer that this should be clarified to mean highly metabolically active and has been changed on line 106.

*Line 104 + 107 - Two sentences begin with "This includes"; one of them could be reworded. E.g., 107 could start with something like "We systematically examined the effects of removal of ..."*

We agree with the reviewer that this duplicate wording should be changed and took the author’s suggestion on line 108.

*End of introduction - A sentence or two at the end of the final paragraph indicating the major take-home message would fit nicely. This could paraphrase the final portion of the Abstract.*

We would like to thank the reviewer for this suggestion and a summary statement has been added to the end of the introduction on lines 109-113.

*Fig 4D, 5F, 6F - Is there a reason why kglc needs to be normalized? If so, indicate this in the legends, or at least indicate that kglc is normalized in the legends.*

The average kglc remains stable across simulations, so in principle this did not need to be normalized. However, we felt that it allowed the reader to more easily compare the kglc in different cell populations to the average value of all cells. The legends for Fig 4,5,6 have been changed to say that kglc is normalized on lines 323, 381, 429.

*Line 655; 665 - "Our results suggest" instead of "suggests"; "properties remain" instead of "remains"*

This was an oversight and has been corrected on lines 668 and 678.

***Reviewer #3:*** *The revised manuscript addresses the previous concerns so that it can be accepted in the current form.*

We thank the reviewer for reviewing our manuscript and recommending it be accepted in its current form.
